# Emotion regulation mediates the relationship between social frailty and stress, anxiety, and depression

**DOI:** 10.1038/s41598-023-33749-0

**Published:** 2023-04-20

**Authors:** Olivia P. Demichelis, Sarah A. Grainger, Ruth E. Hubbard, Julie D. Henry

**Affiliations:** 1 School of Psychology, The University of Queensland, St Lucia, Brisbane, QLD 4072 Australia; 2 Faculty of Medicine, The University of Queensland, Brisbane, Australia

**Keywords:** Psychology, Human behaviour

## Abstract

Social frailty refers to an inability to meet basic social needs and has been identified as a threat to physical and mental health. Although social frailty has been linked with many adverse health and well-being outcomes, potential mediators of the relationship between social frailty and well-being remain poorly understood. Emotion regulation refers to the capacity to alter the experience of emotions to behave in accordance with a desired goal. The present study was designed to provide the first direct test of whether emotion regulation mediates the relationships between social frailty and important well-being outcomes (stress, anxiety, and depression). A total of 790 participants completed validated measures of social frailty, stress, anxiety, depression, and emotion regulation. In line with our preregistered hypotheses, higher social frailty predicted increased stress, anxiety, and depression, and each of these relationships were partially mediated by emotion regulation capacity. These data provide novel evidence that emotion regulation abilities may serve as a protective factor against the negative consequences of social frailty.

## Introduction

Social frailty refers to being at risk of losing, or having lost, important resources that are required to fulfil one’s social needs during the lifespan^1^. It is predictive of many important health and well-being outcomes, including physical frailty^2^, disability^3^, poorer cognitive function, physical and mental health, and even mortality^4,5^. Considering these important consequences of social frailty, it is critical to understand potential factors that might mediate these relationships. This would allow for a more complete understanding of what factors may buffer against these negative consequences of frailty, which could subsequently inform design of future interventions. The present study was designed to provide the first direct test of whether individual differences in the capacity to regulate emotions mediate the relationship between social frailty with stress, anxiety, and depression.

Emotion regulation broadly refers to the ability to modulate the experience of emotions to behave in accordance with a desired goal, as well as one’s awareness, understanding, and acceptance of their emotions^6^. Hence, the absence of any, or all, of these abilities suggest potential difficulties in emotion regulation, or emotion dysregulation. According to the *Process Model of Emotion Regulation,* various strategies can be implemented to regulate an emotion from before its conception to after it has occurred. For example, by selecting or modifying situations that will increase the likelihood of experiencing desirable emotions, or by adjusting one’s appraisal of a situation to alter the impact of an emotion^7^. It seems possible that effective use of emotion regulation might help mitigate the negative effects of social frailty on stress, anxiety, and depression. Indeed, recent evidence shows that greater social frailty is associated with poorer emotion regulation^8^, and expressive suppression, but not cognitive reappraisal^9^. However, to date there has been no test of whether emotion regulation capacity might serve as a mediator in the relationship between social frailty and well-being.

Several distinct models have been proposed that suggest one of the ways in which social frailty might negatively impact well-being is by reducing capacity for emotion regulation. For instance, a central tenet of *Social Baseline Theory*^10–12^ is that humans have evolved to depend on social connections as a foundation for their physical and psychological well-being. Because our social relationships help to maintain a baseline for our emotional states, when individuals experience social frailty, the absence of a strong social foundation means that they have fewer resources to effectively regulate emotional states (i.e., to achieve emotional homeostasis), with negative implications for broader well-being^10–12^. Interestingly, *The Transactional Theory of Stress and Coping*^13,14^ proposes a different but complementary mechanism through which social frailty should influence emotion regulation. This is because, central here too is the idea that social frailty reduces the availability of supportive resources that enable effective management of difficult emotions (in this model, the negative emotions elicited by physiological stress). Thus, based on these theories, it is likely that social frailty negatively impacts well-being through reducing the ability to engage in emotion regulation ability.

Stress, anxiety, and depression are important indicators of well-being that can individually and collectively impact brain, behavior, and cognition (e.g.^15–19^). Higher social frailty has been consistently linked to increased depression^4,8^. Similarly, a separate literature has revealed that specific components of social frailty (social isolation, loneliness, and decreased social support) are related to higher levels of stress and anxiety (e.g.^20–24^). Longitudinal data suggests that the relationships between specific components of social frailty and well-being are bidirectional (e.g.^25–27^). One potential reason for the bidirectional nature of these relationships is that, while social frailty may increase vulnerability to stress, anxiety, and depression because of the associated lack of social relationships or resources to help cope with challenging life events, higher levels of stress, anxiety and depression can also lead to social withdrawal, thereby increasing vulnerability to social frailty.

Emotion regulation is also important in regulating stress, anxiety, and depression. Better emotion regulation capacities are associated with reduced stress^28–31^. Poor emotion regulation capacity has also been found to negatively predict anxiety symptom severity^32^. Indeed, a meta-analysis found that individuals with anxiety disorders demonstrate impaired cognitive reappraisal ability and that this is likely underpinned by hypo-activation of key neural regions required to engage in emotion regulation^33^. Similarly, emotion regulation has been implicated in depression, whereby maladaptive use of emotion regulation strategies is linked with worsening depression^34^. Importantly, emotion regulation has been found to mediate the relationships between other variables and well-being. For instance, it has been found that emotion regulation partially mediates the relationship between physical frailty and depression^9^, mindfulness with symptoms of depression and anxiety^35^, as well as the relationship between sleep with stress^36^. As emotion regulation plays a key role in well-being, it is important to determine if it could also buffer the effects of high social frailty on well-being.

## The present study

This study was designed to provide the first test of whether emotion regulation capacity mediates the relationship between social frailty with stress, anxiety, and depression. As noted, the only prior study to date to test the association between emotion regulation capacity and social frailty found evidence that these were related^8^, and both social frailty and emotion regulation have been consistently linked to important indicators of well-being^28–30,37–39^. Perhaps most importantly, broader research shows that capacity for emotion regulation can be improved via targeted intervention^40,41^, suggesting that emotion regulation interventions may provide a promising avenue for future work focused on trying to ameliorate the negative effects of social frailty on well-being. We predicted that high social frailty would be associated with increased emotion dysregulation, stress, anxiety, and depression. We also predicted that increased emotion dysregulation would be associated with increased stress, anxiety, and depression. Finally, we predicted that emotion regulation capacity would mediate the relationship between social frailty with stress, anxiety, and depression.

## Method

### Transparency

This study was part of a larger study protocol. The pre-registration document, de-identified data, and R file used can be accessed on the Open Science Framework via the following link: https://osf.io/v7k3d/?view_only=383f27b12700410d9fbf86f7d804761f. Ethical approval was provided by the Human Research Ethics Committee at The University of Queensland (Project number: 2021/HE001811). This study was conducted in accordance with relevant guidelines and regulations.

### Participants

This study was part of a larger study protocol^36^. For that study, a power calculation was conducted a priori using G*Power^42^. To be conservative, a minimum of 602 individuals were required to have sufficient power (1 − β > 80%, α = 0.05, two-tailed) to detect a small effect size (f2 = 0.02). A total of 829 participants from the United Kingdom were recruited via the online survey platform Prolific between November 2021 to May 2022. We excluded 39 participants due to technical difficulties or inaccurately answering key variables of interest. This left a total of 790 participants (see Table 1 for demographic characteristics). To be eligible for participation in this study, participants were required to: (1) have high levels of English proficiency; (2) have normal or corrected to normal vision; and (3) have no history of severe head trauma.

**Table 1:** Means, standard deviations, and ranges for key participant demographic variables.

	Mean	*SD*	Range
Age	36.33	14.54	18–81
Gender (F:M:O)	391:372:27	–	–
BMI	31.07	13.22	16–123
Years of education	15.80	2.82	8–30
HADS-A	11.20	4.64	0–21
HADS-D	8.28	4.46	0–20
PSS	23.08	7.88	1–40
DERS	101.01	27.01	39–167
SFI	3.78	1.29	0–8

Income *N* (%)	*N*	%	
< £5,199–£15,599	238	30	–
£15,600–£31,199	208	26	–
£31,200– > £52,000	296	38	–
Prefer not to say	48	6	–

Gender (F:M:O) = Gender of participants (*N* Female: *N* Male: *N* Other or Prefer not to say).

*BMI* body mass index, *HADS-A, D* Hospital Anxiety Depression Scale-Anxiety subscale, Depression subscale, *PSS* Perceived Stress Scale, *DERS* Difficulties in Emotion Regulation Scale, *SFI* Social Frailty Index.

### Procedure

All measures were placed in a single online survey which was uploaded to the *Prolific* platform. Eligibility screening criteria were uploaded with the description of the study. Participants were asked to provide written informed consent at the beginning of the survey and then provide background demographic information. After this, participants completed the key measures, listed below, which were presented in a randomised order (determined by Qualtrics software). The total survey took approximately 35 min to complete. Once participants finished the survey, they were compensated 7.50GBP for their time.

### Key measures

#### Social frailty

The Social Frailty Index (SFI^43^) is an 8-item scale that indexes social frailty. It is a validated measure that assesses access to both general and social resources, as well as the fulfilment of basic social needs. Participants were asked to indicate whether each statement was true for them (Yes = 0, No = 1). Scores were then summed to provide an index of social frailty, whereby higher scores indicated higher social frailty. The internal reliability consistency, measured via McDonald’s omega, was *ω* = 0.66, 95%CI [0.56, 0.73].

#### Stress

The Perceived Stress Scale (PSS^44^) is a validated and widely used measure of the perception of stress. It is a 10-item scale which measures the degree to which situations in an individual’s life are appraised as stressful. The PSS asks participants to rate on a Likert scale ranging from 0 (Never) to 4 (very often) how often they felt or thought a certain way during the past month. Scores were then summed to create a total perceived stress score, whereby higher totals reflected higher levels of perceived stress. The PSS has been validated in both younger and older samples^45,46^. The internal reliability consistency, measured via McDonald’s omega, was *ω* = 0.86, 95%CI [0.74, 0.93].

#### Anxiety and depression

To index anxiety and depression, the Hospital Anxiety & Depression Scale (HADS^47^) was used. The HADS is a self-report scale comprising seven items that assess anxiety and seven items that assess depression. Participants were asked to rate on a scale from 0 to 3 which statement was closest to how they have been feeling in the past week. Scores were then totalled for each subscale with higher scores indicating higher levels of anxiety and depression. The internal reliability consistency, measured via McDonald’s omega, for the anxiety and depression subscales were *ω* = 0.89, 95%CI [0.85, 0.91] and *ω* = 0.86, 95%CI [0.85, 0.91], respectively.

#### Emotion regulation

The Difficulties in Emotion Regulation Scale (DERS^6^) is a validated 36-item questionnaire assessing deficits in emotion regulation. The statements of behaviour or cognition are categorised into six subscales: (1) nonacceptance of emotional responses, (2) difficulties in engaging in goal-directed behaviours, (3) impulse control difficulties, (4) lack of emotional awareness, (5) limited access to emotion regulation strategies, and (6) lack of emotional clarity. Participants were asked to rate the extent to which each statement was true for them on a scale from 1 (almost never) to 5 (almost always). Scores were then totalled to create an index of difficulty in emotion regulation, whereby higher scores indicated worse emotion regulation difficulties. This measure has been validated in broad community lifespan samples^48,49^. The internal reliability consistency, measured via McDonald’s omega, for all subscales were as follows: Awareness *ω* = 0.87, 95%CI [0.88, 0.94]; Clarity* ω* = 0.91, 95%CI [0.89, 0.92]; Impulse *ω* = 0.91, 95%CI [0.90, 0.95]; Nonacceptance *ω* = 0.95, 95%CI [0.94, 0.96]; Goals *ω* = 0.91, 95%CI [0.90, 0.94]; Strategies *ω* = 0.93, 95%CI [0.92, 0.94].

### Statistical analyses

Little’s MCAR was conducted on the key variables of interest (PSS, HADS, DERS, and SFI) using IBM SPSS Statistics version 27.0 for Windows. Results suggested that the missing data could not be predicted by another variable in the data set *χ*^2^(249) = 178.520, *p* = 1.000. As only 10 participants were missing data, and each were missing over 50% of their responses, all data for these participants were deleted. All remaining analyses were conducted in R version 4.1.0. Pearson correlations were conducted between social frailty with stress, anxiety, depression, and emotion regulation. Three separate mediation analyses were performed using the PROCESS Macro^50^, where social frailty was the independent variable, difficulties in emotion regulation was the mediator variable, and stress, anxiety, and depression were the dependent variables. We conducted a non-parametric bootstrapping procedure to compute confidence intervals (CIs) around the indirect effect. The final estimate of the indirect effect is represented by the mean indirect effect computed across 5000 bootstrap samples.

## Results

### Correlations

Higher social frailty was associated with higher levels of stress, anxiety, and depression, and worse emotion regulation. Higher levels of stress, anxiety, and depression were also associated with worse emotion regulation (see Table 2; see Fig. S1 for scatterplots depicting the correlations).

**Table 2:** Pearson correlations between key variables.

	SFI	DERS	PSS	HADS-A
SFI				
DERS	0.31**			
PSS	0.37**	0.71**		
HADS-A	0.29**	0.63**	0.76**	
HADS-D	0.41**	0.58**	0.66**	0.60**

*SFI* Social Frailty Index, *DERS* Difficulties in Emotion Regulation Scale, *PSS* Perceived Stress Scale, *HADS-A/D* Hospital Anxiety and Depression Scale-Anxiety/Depression subscale.

** indicates *p* < .01.

### Mediations

**Figure 1:**
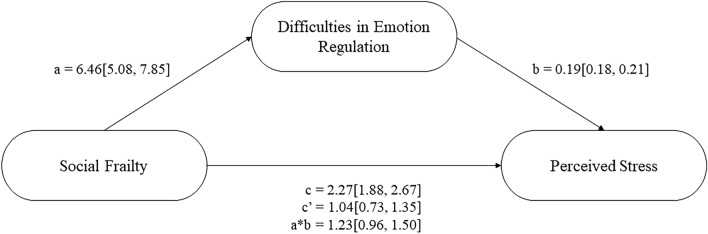
Difficulties in emotion regulation as a partial mediator between social frailty and perceived stress.

**Figure 2:**
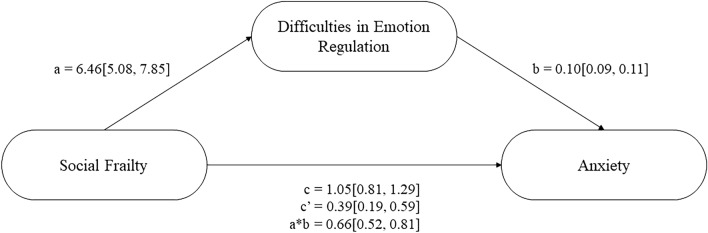
Difficulties in emotion regulation as a partial mediator between social frailty and anxiety.

**Figure 3:**
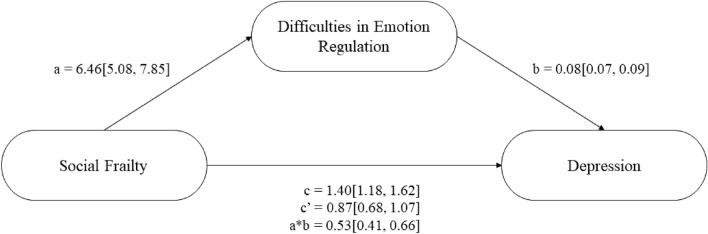
Difficulties in emotion regulation as a partial mediator between social frailty and depression.

The next step in analyses was to conduct a series of mediation analyses (see Figs. 1, 2, and 3 respectively). These analyses revealed that higher social frailty predicted greater emotion dysregulation, which predicted increased stress, anxiety, and depression (see Table 3). The direct effect, and indirect effect with percentile bootstrapping, for each model were significant, suggesting that higher social frailty directly and indirectly predicted increased stress, anxiety, and depression (see Table 3). Approximately 52% of the variance in stress was accounted for by the predictors (social frailty and emotion regulation; *R*^2^ = 0.52, *F*(2, 787) = 432.560, *p* < 0.001). Approximately 41% of the variance in anxiety was accounted for by the predictors (*R*^2^ = 0.41, *F*(2, 787) = 270.38, *p* < 0.001). Approximately 39% of the variance in depression was accounted for by the predictors (*R*^2^ = 0.39, *F*(2, 787) = 251.21, *p* < 0.001).

**Table 3:** Unstandardised regression coefficients, standard error and 95% confidence intervals for each mediation model.

Relationship	*B*	*SE*	*p*-value	Lower CI	Upper CI
Social frailty and stress mediation					
SFI on DERS	6.46	0.71	< 0.001	5.08	7.85
DERS on PSS	0.19	0.01	< 0.001	0.18	0.21
Direct effect	1.04	0.16	< 0.001	0.73	1.35
Indirect effect	1.23	0.14	–	0.97	1.50
Social frailty and anxiety mediation					
SFI on DERS	6.46	0.71	< 0.001	5.08	7.85
DERS on HADS-A	0.10	0.01	< 0.001	0.09	0.11
Direct effect	0.39	0.10	< 0.001	0.19	0.59
Indirect effect	0.66	0.08	–	0.52	0.81
Social frailty and depression mediation					
SFI on DERS	6.46	0.71	< 0.001	5.08	7.85
DERS on HADS-D	0.08	0.01	< 0.001	0.07	0.09
Direct effect	0.87	0.10	< 0.001	0.68	1.07
Indirect effect	0.53	0.06	–	0.41	0.66

The indirect effect for each mediation used Bootstrapping.

*CI* confidence interval, *SFI* Social Frailty Index, *DERS* Difficulties in Emotion Regulation Scale, *PSS* Perceived Stress Scale, *HADS-A/D* Hospital Anxiety and Depression Scale-Anxiety/Depression subscale.

## Discussion

This study provides a clearer understanding of the role of emotion regulation in understanding the relationship between social frailty and well-being. In line with our predictions higher social frailty was associated with worse emotion regulation, and higher stress, anxiety, and depression. Similarly, poorer emotion regulation was associated with increased stress, anxiety, and depression. Importantly, the present study meaningfully extends on the prior literature by demonstrating for the first time that difficulties in emotion regulation partially mediated the relationship between social frailty with stress, anxiety, and depression. Although preliminary, these data provide initial evidence that a greater capacity for emotion regulation may be an important buffer against the negative effects of social frailty on well-being and could be a potential target for future interventions focused on reducing the negative effects of social frailty on stress, anxiety, and depression.

The results from this study align with the findings from the broader literature. As noted, social frailty—or specific components of social frailty—has been linked to increased emotion dysregulation^8,9^, depression^4,8^, and stress and anxiety^20–24^. Furthermore, increased emotion dysregulation has been consistently linked to increased stress, anxiety, and depression^28–30,34,37–39^. This study therefore provides further evidence to suggest that higher social frailty is associated with increased stress, anxiety, and depression. Our results also align with prior literature that has found that emotion regulation mediated the relationships between other variables with well-being^35,36^. Importantly, this is the first study to date to demonstrate that emotion regulation may provide a buffer for the effects of social frailty on well-being.

It is important to highlight that the social frailty and well-being relationships are likely bidirectional, whereby longitudinal studies have demonstrated that specific components of social frailty can lead to poorer well-being outcomes, and vice versa^25–27^. It is possible that the former relationship occurs because higher social frailty could increase vulnerability to stress, anxiety, and depression by reducing social resources that assist in coping with challenging life events. Higher levels of stress, anxiety, and depression could also result in an individual withdrawing socially from their support networks. The present results provide support for the pathway that individuals with high levels of social frailty may be particularly vulnerable to poorer mental health outcomes. Further research is therefore required to examine the potential bidirectional nature of these relationships.

Increased social frailty has been found to be predictive of poor health and well-being outcomes^2–5^. Although further research is needed, and in particular studies that use longitudinal designs, the results of both the current study and prior literature linking social frailty to poor well-being outcomes^4,8,20–24^, have potential implications for how intervention efforts could be targeted. Namely, interventions that function to strengthen emotion regulation might be of particular benefit for people with high social frailty who struggle with stress, anxiety, and/or depression.

## Limitations and future directions

The present study had several notable strengths, including a large, well characterised sample. We also attempted to recruit a cohort that was broadly representative in terms of key demographics. However, some limitations need to be noted. As with all cross-sectional research designs, an important limitation of this study that needs to be acknowledged is that it was not possible to infer causality. Because prior literature suggests that conducting mediation analyses with cross-sectional data can increase the risk of biased estimates when interpreting potential longitudinal causal pathways^51^, an important next step in this literature is to use longitudinal paradigms assessing these variables over time to infer causality and potential bidirectionality. Similarly, it would be helpful to assess these mediational relationships in groups of individuals with high levels of social frailty to determine how well emotion regulation capacities can buffer the effects of social frailty on well-being in severely socially frail individuals.

Although this study used extensively validated measures of social frailty, stress, anxiety, depression, and emotion regulation, further research is required to determine how robust the findings are by replicating the results across different measures of these constructs. In particular, an important limitation of this work that needs to be acknowledged is that all of the measures used were self-report, which are susceptible to response biases. Indeed, recent work has shown that self-report may not capture emotion regulation capacity as it occurs in daily life^52^. Future studies are therefore now needed to test whether capacity for emotion regulation continues to mediate these relationships when it is indexed using other methods. For instance, experimental paradigms where participants are explicitly instructed to, or spontaneously, engage different types of regulation strategy in response to emotionally-evocative stimuli^53^ or ecological momentary assessment which captures emotion regulation in daily life^52^. In addition, while stress, anxiety, and depression are three important aspects of well-being, it remains to be established whether emotion regulation would mediate the relationship between social frailty and other indicators of this broad construct (such as happiness or physical health). Finally, because this study only assessed social frailty, an important next step in this literature is to establish whether emotion regulation also mediates the relationship between well-being with both physical frailty as well as cognitive frailty.

## Conclusion

These data provide further evidence that social frailty and well-being are strongly related. They also highlight a potentially important role for emotion regulation in protecting against the negative effects of social frailty on stress, anxiety, and depression. Further longitudinal studies are the next important step to directly test the directionality of these effects, and to establish whether emotion regulation should be a specific target for future interventions aimed at reducing the negative effects of social frailty.

## Supplementary Information

Supplementary Figure S1.

## Data Availability

The pre-registration document, de-identified data, and R file used can be accessed on the Open Science Framework via the following link: https://osf.io/v7k3d/?view_only=383f27b12700410d9fbf86f7d804761f.
