# Supplementary material for: Emotion regulation mediates the relationship between social frailty and stress, anxiety, and depression
Source: Sci Rep. 2023 Apr 20;13:6430. doi: 10.1038/s41598-023-33749-0 (PMC10119122; doi:10.1038/s41598-023-33749-0)
Supplement: Supplementary file 1 — Supplementary Figure S1. [file 41598_2023_33749_MOESM1_ESM.docx]

**Emotion regulation mediates the relationship between social frailty and stress, anxiety, and depression**

**Supplementary file**

**
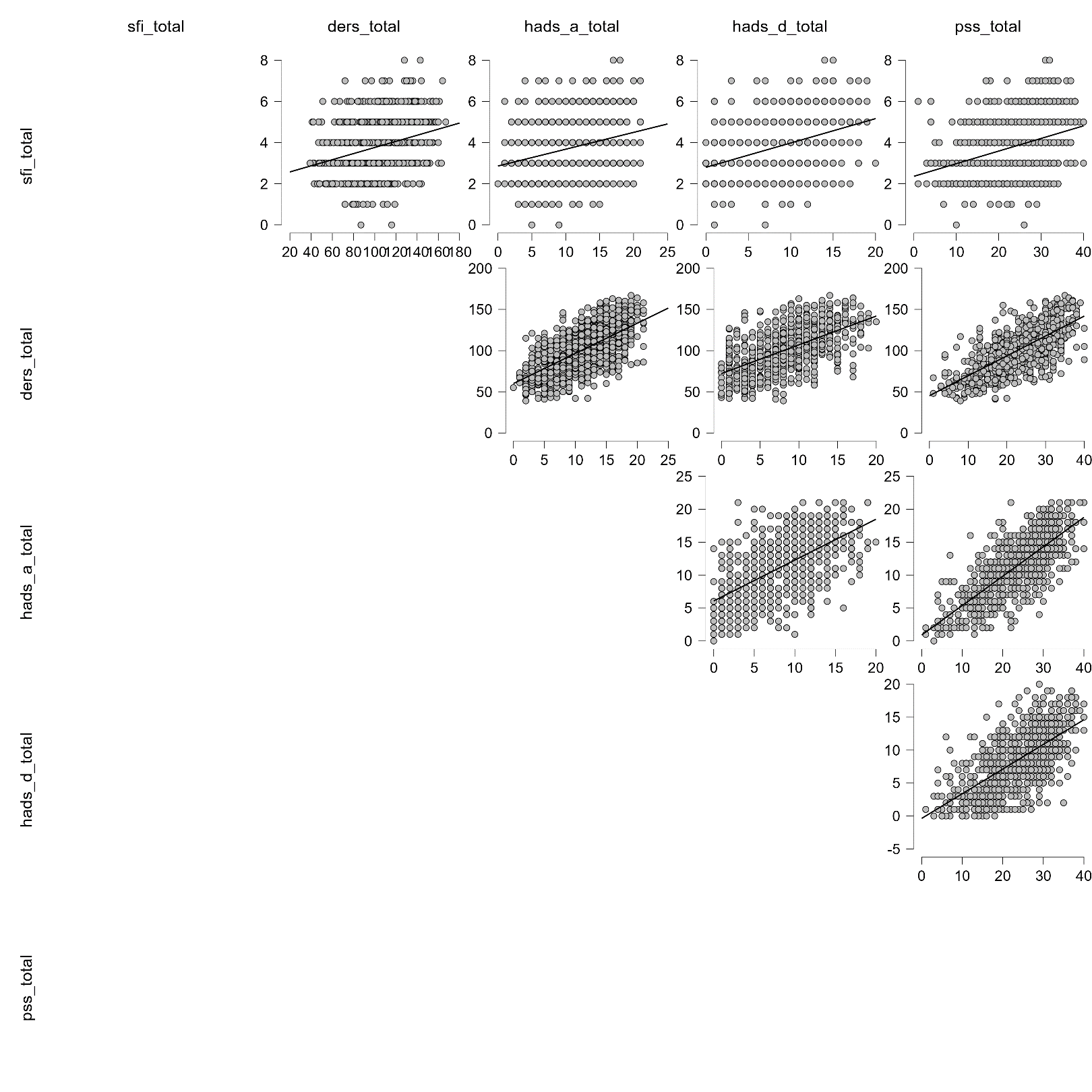
Figure S1. Scatterplots depicting correlations between key variables.**
